# Differing Complex Microbiota Alter Disease Severity of the IL-10^−/−^ Mouse Model of Inflammatory Bowel Disease

**DOI:** 10.3389/fmicb.2017.00792

**Published:** 2017-05-11

**Authors:** Marcia L. Hart, Aaron C. Ericsson, Craig L. Franklin

**Affiliations:** ^1^Comparative Medicine Program, Department of Veterinary Pathobiology, University of MissouriColumbia, MO, USA; ^2^Department of Veterinary Pathobiology, University of MissouriColumbia, MO, USA; ^3^University of Missouri Metagenomics Center, University of MissouriColumbia, MO, USA; ^4^Mutant Mouse Resource and Research Center, University of MissouriColumbia, MO, USA

**Keywords:** gut microbiota (GM), complex microbiota targeted rederivation (CMTR), inflammatory bowel disease, IL-10^−/−^ mouse model, 16S rRNA V4, next generation sequencing, disease phenotype, mouse models

## Abstract

It is estimated that 1.4 million people in the United States suffer from Inflammatory Bowel Disease (IBD), with an overall annual health care cost of more than $1.7 billion. Although the exact etiology of this disease remains unknown, research suggests that it is a multifactorial disease associated with aberrant gastrointestinal microbial populations (dysbiosis). The C57BL/6 and C3H/HeJBir mouse strains with targeted mutations in the IL-10 gene are commonly used models to study IBD. However, anecdotally, disease phenotype can vary in severity from lab to lab. Moreover, studies using germfree and monocolonized mice have suggested that gut microbiota (GM) are critical to disease induction in these models. With recent studies suggesting variation in naturally occurring GM composition and complexity among mouse producers, we hypothesized that differences in these naturally occurring complex GM profiles may modulate disease severity in the IL-10^−/−^ mouse model. To test this hypothesis, we use a technique referred to as complex microbiota targeted rederivation (CMTR) to transfer genetically identical C57BL/6 IL-10^−/−^ and C3H/HeJBir IL-10^−/−^ embryos into surrogate CD-1 or C57BL/6 dams from different commercial producers with varying microbiota complexity and composition. We found that disease severity significantly and reproducibly differed among mice in both IL-10^−/−^ strains, dependent on differing maternally inherited GM. Furthermore, disease severity was associated with alterations in relative abundance of several physiologically relevant bacterial species. These findings suggest that the composition of the resident GM is a primary determinant of disease severity in IBD and provide proof-of-concept that CMTR can be used to investigate the contribution of contemporary complex GM on disease phenotype and reproducibility.

## Introduction

Inflammatory bowel disease (IBD) is a multifactorial disease thought to involve a complex interaction between genetic and environmental factors, leading to aberrant immune responses to normally innocuous microbes (Zhang and Li, 2014; de Souza and Fiocchi, 2016). Two known primary forms of disease exist, ulcerative colitis, and Crohn's disease (CD), which share many clinical symptoms and are characterized by chronic, relapsing inflammation affecting the gastrointestinal (GI) tract. Both forms of IBD are associated with substantial morbidity and long-term costs to patient, health care, and society (Kappelman et al., 2008, 2013). CD preferentially occurs in the distal ileum and colon, areas that contain the highest density of bacteria, suggesting that alterations in bacterial communities (dysbiosis) within these regions may influence disease outcome. Although the exact etiology of IBD remains unknown, recent advances in sequencing technology have shown that patients with IBD have an abnormal composition and function of GI microbiota (Bibiloni et al., 2006; Frank et al., 2007). Further demonstrating the role of the gut microbiota (GM) in disease is the positive clinical response of some IBD patients when antimicrobials and probiotics are prescribed (Sartor, 2004, 2008). However, despite considerable progress in the study of IBD, the mechanism of how changes in microbial diversity and composition induce disease or alter disease outcome remains largely unknown.

Studies of human IBD patients have contributed greatly to the understanding of IBD. However, these studies are largely retrospective and can be confounded by experimental variables such as variation in genetics, environment, and patient compliance. Mouse models that mimic human IBD have also contributed extensively to our understanding of disease pathogenesis as they allow experimentation with tightly controlled variables. A number of widely used models utilize chemical induction of inflammation via administration of epitheliotoxic compounds such as dextran sulfate sodium (DSS), or genetic modification that lead to the development of chronic inflammation (e.g., IL-10 deficient mice; Wirtz and Neurath, 2007). These models can also be used to study the role of the microbiota in IBD, with most options focused on the use of germfree, monocolonized, or defined microbiota animals. While these models are largely useful for the study of specific GM populations, they are by definition restricted in GM complexity and do not fully recapitulate the complexity of the human GM.

Helicobacter-inoculated IL-10^−/−^ mice provide an attractive animal model in which to study the role of complex GM communities, for several reasons. First, while inoculation with *H. hepaticus* is required for disease induction, monocolonization with *H. hepaticus* does not cause inflammation, even in genetically susceptible hosts such as IL-10-deficient mice (Dieleman et al., 2000). Rather, a background population of resident microbes is required (Sellon et al., 1998). The closely related enterohepatic species of helicobacter, *H. bilis*, functions as a provocateur of host immune responses against resident microbes, presumably by causing a transient decrease in epithelial barrier function (Jergens et al., 2006, 2007). A similar effect of *H. hepaticus* is suggested by the decreased expression of tight junction proteins in strains of mice susceptible to *H. hepaticus*-induced inflammation as compared to strains that are colonized but resistant to *H. hepaticus*-induced inflammation (Myles et al., 2007). While recent reports of differential disease severity in IL-10^−/−^ mice maintained at different institutions suggest that factors related to the GM could be responsible for enhancing (or diminishing) inflammation, these studies were not completely controlled (Yang et al., 2013). As such, the observed differences in disease could be due to genetic drift or other institution-dependent factors.

Recent studies suggest that mice from varying vendors can differ in both GM composition and complexity (Hildebrand et al., 2013; Ericsson et al., 2015b). In the current study, we sought to exploit these differences and use rederivation by embryo transfer as a means to study the effect of naturally-occurring complex GM profiles in isogenic animals on disease severity. As this method purposefully focuses on transfer of GM by rederivation, we have referred to this technique as complex microbiota targeted rederivation (CMTR). Specifically, genetically identical C57BL/6 IL-10^−/−^ and C3H/HeJBir IL-10^−/−^ embryos were transferred into surrogate CD-1 or C57BL/6 dams harboring distinct complex gut microbial communities. Pups were inoculated with *Helicobacter hepaticus* at 24 and 26 days of age and colonic and cecal disease severity was assessed by histopathology at 111 days of age. Alterations in GM composition and complexity was evaluated by microbial 16S rRNA amplicon sequencing respectively. We found that disease severity was significantly decreased in mice colonized with the GM derived from mice from Charles River Laboratories (GMCRL) as compared to mice colonized with the GM from The Jackson Laboratory (GMJAX) or Taconic Bioscience (GMTAC). Furthermore, several physiologically relevant bacterial species were found to be altered in all groups dependent on which surrogate dam was used for rederivation.

## Materials and methods

### Generation of B6 and C3H IL-10^−/−^ mice with B6 JAX, B6 TAC, or CD1 CRL gut microbiota

#### Mice

For embryo transfer (ET) recipients, 8–10 week old female C57BL/6J (The Jackson Laboratory, Bar Harbor, ME), C57BL/6NTac (Taconic Biosciences, Inc., Cambridge, IN facilities), and Crl:CD1 (Charles River Laboratories, Wilmington, MA) mice were purchased and allowed to acclimate for 1 week prior to use. Embryos from 8 week-old female B6.129P2-Il10^*tm*1*Cgn*^/J (B6 IL-10^−/−^) and C3Bir.129P2(B6)-Il10^*tm*1*Cgn*^/J (C3H IL-10^−/−^; The Jackson Laboratory) mice were harvested from colonies maintained on site. Vasectomized, 8–10 week old Crl:CD1 male mice (Charles River Laboratories) were co-housed to induce pseudopregnancy and intrauterine embryo transfer was performed (see section Embryo Collection and Transfer). All mice were housed in microisolator cages on ventilated racks (Thoren, Hazelton, PA) on a 14:10 light dark cycle, and provided *ad libitum* access to 5,058 irradiated breeder chow (LabDiet, St. Louis, MO) and acidified autoclaved water.

#### Embryo collection and transfer

On day 1, B6.129P2-Il10^*tm*1*Cgn*^/J, C3Bir.129P2(B6)-Il10^*tm*1*Cgn*^/J, or Crl:CD1 embryo donors received IP injection of 5 IU of pregnant mare serum gonadotropin (PMSG) (Calbiochem, San Diego, CA) in 0.2 ml Dulbecco's phosphate-buffered saline (DPBS) with no calcium or magnesium (Life Technologies, Carlsbad, CA) at 2.5 h post-light induction to induce superovulation. On day 3, at 5 h post-light induction, embryo donors received an IP injection of 5 IU human gonadotropin (hCG) in 0.2 ml DPBS and were mated to intact males of the same genotype. Post-mating, B6.129P2-Il10^*tm*1*Cgn*^/J, C3Bir.129P2(B6)-Il10^*tm*1*Cgn*^/J, or Crl:CD1 embryo donors were euthanized and embryos were collected aseptically. Briefly, the peritoneal cavity was opened and the reproductive tract visualized. Oviducts were excised and placed in 50 μl of pre-warmed type IV-S hyaluronidase (Sigma, St. Louis) reconstituted at 1 mg/ml in HEPES media (Sigma) supplemented with 4 mg/ml bovine serum (Sigma) for 5–10 min. Clutches of embryos were released from oviducts with gentle manipulation under a dissecting microscope, and collected with a sterile glass hand-pipette. In preparation for embryo transfer, surrogate embryo recipient females (Crl:CD1, Charles River Laboratories; C57BL/6J, The Jackson Laboratory; C57BL/6NTac, Taconic Biosciences, Inc., Cambridge, IN facilities) demonstrating signs of estrus were mated with a sterile, vasectomized Crl:CD1 male (Charles River Laboratories). Following mating, surrogate females were inspected for copulatory plugs and plug-positive mice were used for embryo transfer. For the latter, surrogate females were anesthetized via IM injection of ketamine/xylazine cocktail at 5.5 mg and 1 mg per 100 g body weight respectively, and placed in sternal recumbency. A dorsal midline incision was made and the uterine oviducts located by dissecting through the retroperitoneal muscle. Embryos in 3–5 μl of media were injected into the oviducts using a glass hand-pipette. Skin incisions were closed with sterile surgical staples and mice received a subcutaneous injection of 2.5 mg/kg of body weight flunixin meglumine (Banamine®) prior to recovery on a warming pad.

### Generation of outbred CD1 mice with B6 JAX, B6 TAC, or CD1 CRL gut microbiota and subsequent rederivation of B6 IL-10^−/−^ mice

We also sought to establish colonies of outbred mice harboring distinct complex microbiota that is naturally occurring in contemporary rodent producers. Doing so would facilitate future CMTR studies as outbred mice have greatly improved reproductive indices. To this end, 8–10 week old female C57BL/6J (The Jackson Laboratory), C57BL/6NTac (Taconic Biosciences, Inc.), and Crl:CD1 (Charles River Laboratories) mice were purchased and allowed to acclimate for 1 week prior to use. Embryos were harvested from 8 week old Crl:CD1 (Charles River Laboratories) mice and implanted into recipient C57BL/6J (The Jackson Laboratory), C57BL/6NTac (Taconic Biosciences, Inc.), and Crl:CD1 (Charles River Laboratories) mice as previously described section Embryo Collection and Transfer. Offspring from surrogate dams were mated using an outbred mating scheme and individual colonies with targeted GM were maintained for two generations. Second generation 8–10 week old females were used for rederivation of B6.129P2-Il10^*tm*1*Cgn*^/J (B6 IL-10^−/−^) mice.

### Bacterial cultivation and inoculation

*Helicobacter hepaticus* (MU94) was grown on 5% sheep blood agar plates (Becton Dickinson, Franklin Lakes, NJ) overlaid with 5 ml *Brucella* broth (Becton Dickinson) supplemented with 5% fetal bovine serum (Sigma-Aldrich, St. Louis, MO) and incubated for 24 h at 37°C in a microaerobic chamber with 90%N_2_, 5%H_2_, and 5%CO_2_ gas mixture. Twenty-four hours later, cultures were transferred to 250 ml Erlenmeyer flasks containing *Brucella* broth supplemented with 5% fetal bovine serum and incubated for an additional 48 h. Immediately prior to use, cultures were observed microscopically for viability. Experimental mice were intra-gastrically gavaged at 23 and 25 days of age with 10^8^ bacteria suspended in 0.5 ml sterile *Brucella* broth.

### qPCR of *Helicobacter hepaticus*

DNA was extracted from cecal contents to determine the relative abundance of *H. hepaticus* in rederived mice using qPCR analysis and previously described *Helicobacter hepaticus* primers (Beckwith et al., 1997). Briefly, samples were assayed in triplicate in 10 μL reactions that each contained 0.25 μL of each primer, 5 μL QuantiTect SYBR Green PCR Master Mix (BioRad, Hercules, CA), 2.75 μL nuclease-free water, and 2 μL sample DNA. Reactions were amplified in a real-time thermocycler (C1000 Touch Thermal Cycler CFX384 Real Time system, BioRad) using the following parameters: incubation at 95°C for 15 min, 15 s of denaturation at 94°C (40 cycles), 20 s of annealing at 58°C, and 30 s of extension at 72°C. Fluorescence was monitored at the end of each extension phase (BioRAD CFX Manageer 3.1, Biorad). Technical replicates were averaged and fold change of each GM group was compared to *H. hepaticus* expression in the GMCRL group.

### Evaluation of cecal and colonic disease

At 111 ± 2 days of age, experimental mice were euthanized by CO_2_ asphyxiation, and necropsy was performed. The entire gastrointestinal tract was removed and the colon length from cecal attachment to anus was measured. The cecum was incised longitudinally, and cecal contents were removed and placed in a sterile 2 ml round bottom tube. Samples were flash frozen in liquid nitrogen and stored at −80°C until processed for DNA extraction. Cecal tissue was rinsed with sterile saline and laid flat, serosal surface down, on a small section of white note card. The colon was removed and rinsed with sterile saline. Tissues were immersed in 10% buffered formalin for 48 h and processed for hematoxylin and eosin staining. Histological evaluation was performed by a rodent pathologist, blinded to the identity of the animal's experimental group.

### Scoring of cecal and colonic lesions

Tissues were evaluated for disease using a scoring system previously adapted by our lab (Alvarado et al., 2015). Detailed evaluation of the severity of epithelial hyperplasia (0, none; 1, mild; 2, moderate, 3, severe with crypt branching, and/or herniation; 4, dysplasia) and inflammatory changes (0, no inflammation 1, mild inflammation limited to the mucosa; 2, moderate inflammation limited to the mucosa and submucosa; 3, severe inflammation with obliteration of normal architecture, erosions, crypt abscesses; and 4, severe inflammatory changes with ulceration). In addition, a separate score for the longitudinal extent of epithelial hyperplasia and inflammation was assigned (0, no significant changes; 1, one or two foci occupying <10% of the mucosa; 2, multifocal lesions occupying 10–60% of the mucosa; 3, diffuse lesions occupying >60% of the mucosa). The final lesion score was calculated as (hyperplasia score × longitudinal extent of hyperplasia score) + (inflammation score × longitudinal extent of inflammation score). Total disease scores ranged from 0 (no disease) to 24 (severe disease).

### Isopropanol DNA extraction of cecal contents

DNA extraction was performed as previously described (Ericsson et al., 2015b). Briefly, cecal contents were thawed at room temperature and a sterile 0.5 cm diameter stainless steel bead and 800 μl of lysis buffer were added to the 2 ml round-bottom tube. Samples were mechanically disrupted using a TissueLyser II (Qiagen, Venlo, Netherlands) for 3 min at 30 Hz, followed by incubation at 70°C for 20 min with periodic vortexing. Samples were centrifuged at 5,000 × g for 5 min, and the supernatant was transferred to a sterile 1.5 ml Eppendorf tube containing 200 μl of 10 mM ammonium acetate. Lysates were vortexed, incubated on ice for 5 min, and then centrifuged. Supernatant was transferred to a sterile 1.5 ml Eppendorf tube and one volume of chilled isopropanol was added. Samples were incubated on ice for 30 min and centrifuged at 16,000 × g at 4°C for 15 min. The DNA pellet was washed with 70% ethanol and resuspended in 150 μl Tris-EDTA, followed by addition of 15 μl of proteinase K and 200 μl AL buffer (DNeasy Blood and Tissue kit, Qiagen). Samples were incubated at 70°C for 10 min and 200 μl of 100% ethanol was added to the tubes. Samples were mixed by gentle pipetting and the contents transferred to a spin column from the DNeasy kit (Qiagen). The DNA was further purified following the manufacturer's instructions and eluted in 200 μl EB buffer (Qiagen). DNA concentrations were determined fluorometrically (Qubit dsDNA BR assay, Life Technologies, Carlsbad CA) and samples were stored at 20°C until sequencing.

### Library construction and 16S rRNA sequencing

Library construction and sequencing was performed at the University of Missouri DNA Core. Bacterial 16SrRNA amplicons were generated in a multiplexed (96-well) format using amplification of the V4 hypervariable region of the 16S rRNA gene, and then sequenced on the Illumina MiSeq platform as previously described (Ericsson et al., 2015b). Samples returning >10,000 reads were deemed to have successful amplification.

### Informatics analysis

Assembly, binning, and annotation of DNA sequences were performed at the University of Missouri Informatics Research Core Facility. Briefly, contiguous DNA sequences were assembled using FLASH software (Magoc and Salzberg, 2011), and culled if found to be short after trimming for a base quality <31. Qiime v1.8 (Kuczynski et al., 2011) software was used to perform *de novo* and reference-based chimera detection and removal, and remaining contiguous sequences were assigned to operational taxonomic units (OTUs) using a criterion of 97% nucleotide identity. Taxonomy was assigned to selected OTUs using BLAST (Altschul et al., 1997) against the Greengenes database (DeSantis et al., 2006) of 16SrRNA sequences and taxonomy.

### Statistical analysis

Statistical analysis was performed using Sigma Plot 13.0 (Systat Software Inc., Carlsbad CA). Differences in lesion score, phylum, and OTU relative abundance were determined using two-way ANOVA with sex and GM profile (i.e., GMJAX, GMTAC, GMCRL) as the independent variables, followed by Student Newman-Keuls *post hoc* test. To account for multiple testing, OTUs with a *p* < 0.01 were considered statistically significant. Statistical differences in Shannon index, colon length, and relative abundance of *H. hepaticus* following quantitative PCR were first evaluated using two-way ANOVA to assess main effects and interactions between sex and GM profile. No main effects were detected between male and female mice in any of those values, thus data from male and female mice were pooled and all subsequent analyses was performed using one-way ANOVA with Student Newman-Keuls *post hoc* test. Tests with a *p* < 0.05 were considered statistically significant. For gut microbiota analysis, OTUs with <10,000 reads were excluded from the data set. Bar graphs were generated with Microsoft Excel (Microsoft, Redmond WA) and principal component analysis (PCA) was generated using Paleontological Statistics Software Package (PAST) 3.12 (Hammer et al., 2001). All groups were visually inspected for descriptive analysis of consistency between animals (bar graphs) or clustering of animals within groups (PCAs). Statistical testing for differences in beta-diversity was performed via PERMANOVA, implemented using PAST 3.12.

## Results

### IL-10^−/−^ mice with GMCRL have decreased typhlocolitis severity

**Figure 1:**
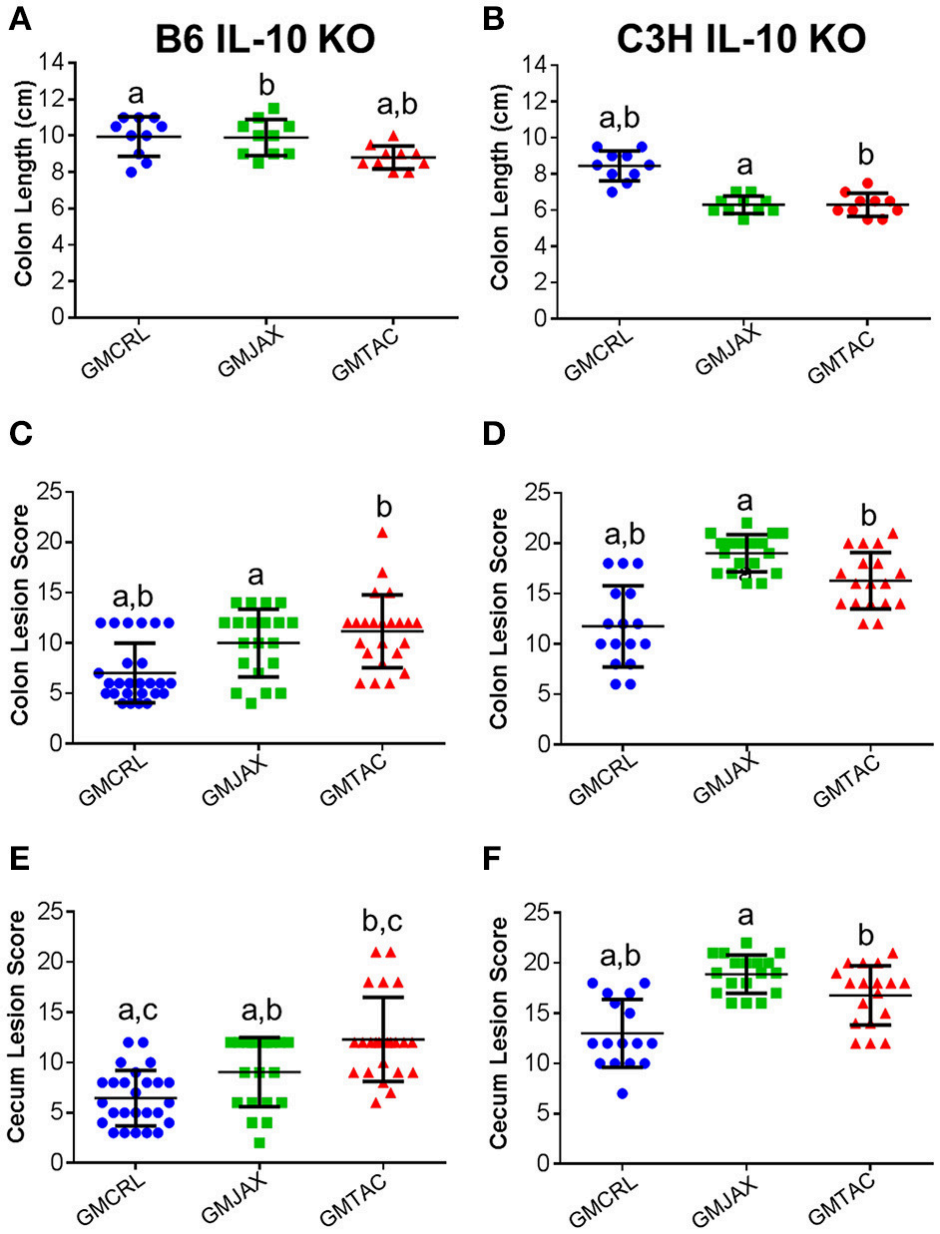
**Colonic and cecal disease is decreased in male and female mice rederived to GMCRL surrogate dams**. Colon length of B6 **(A)** and C3H **(B)** IL-10^−/−^ mice with differing gut microbiota profiles: GMCRL (Charles River Laboratories), GMJAX (The Jackson Laboratory), and GMTAC (Taconic); (*n* = 10 per group). Colon lesion score of B6 **(C)** and C3H **(D)** IL-10^−/−^ mice with differing GM profiles (*n* = 15–25 per group). Cecal lesion score of B6 **(E)** and C3H **(F)** IL-10^−/−^ mice with differing GM profiles (*n* = 15–25 per group). Colon length measured at time of necropsy from cecum to anus. Statistical significance determined by one way-ANOVA with Student Newman-Keuls *post-hoc* test. *p* ≤ 0.05 is significant. Colonic and cecal lesion scores based on severity and longitudinal extent of epithelial hyperplasia and inflammation (0 = no disease and 24 = most severe disease). Statistical significance of lesion scores and sex determined using two way-ANOVA with Student Newman-Keuls *post-hoc* test. Bars indicate mean and SEM. *p* ≤ 0.05 is significant. Statistical significance between groups annotated by same lower case letters above dot plots.

In order to determine the impact of differing complex GM communities on disease severity, B6 IL-10^−/−^ embryos were transferred to surrogate dams purchased from three different vendors (Figure S1). Surrogate dams were allowed to naturally deliver and raise pups. To induce disease, weanling pups were inoculated with *Helicobacter hepaticus* at 24 and 26 days of age. Experimental mice were euthanized at 111 days of age and the cecum and colon were removed for gross evaluation of colon length and histopathologic assessment of disease severity. Grossly, mice rederived in surrogate dams harboring Taconic GM (hereafter referred to as GMTAC) had significantly decreased colon length when compared to mice rederived in surrogate dams with either GMCRL (Charles River Laboratories) or GMJAX (The Jackson Laboratory) microbiota profiles (Figure 1A). To further evaluate disease severity, histopathologic scoring of lesions in the colon and cecum was performed. Rederived B6 IL-10^−/−^ mice harboring GMCRL had significantly lower mean lesion scores in both the colon (Figure 1C) and cecum (Figure 1E) as compared to mice colonized with GMJAX and GMTAC. In addition, mice rederived to surrogate dams with GMJAX had lower cecal lesion scores than mice rederived to surrogate dams with GMTAC. No differences in lesion severity were detected between male and female mice in any of the GM groups examined.

To determine if these findings were unique to the IL-10^−/−^ model on a C57BL/6 genetic background, C3H IL-10^−/−^ embryos were implanted in surrogate dams from the same vendors as used in the B6 IL-10^−/−^ experiment. In the C3H IL-10^−/−^ model, mice rederived in surrogate dams with either GMJAX or GMTAC had significantly shorter colon length than mice rederived in surrogate dams with GMCRL (Figure 1B), while no statistical difference in colon length was found between the GMJAX and GMTAC groups. As in the B6 genetic background, mice rederived in surrogate dams with GMCRL had significantly lower mean lesion scores in both the colon (Figure 1D) and cecum (Figure 1F) than either the GMJAX or GMTAC groups, with differences in mean lesion score also detected between mice harboring GMJAX and GMTAC. Again, no differences were found in lesion severity between males and females in the GM groups examined.

### Diversity and composition of GM profiles differ following disease onset

**Figure 2:**
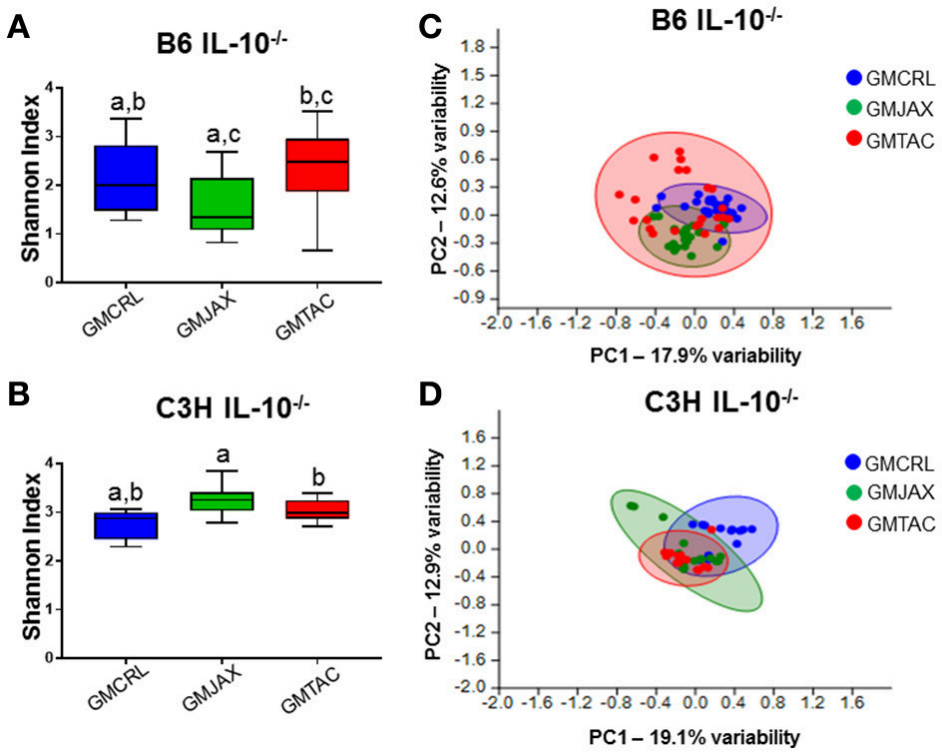
**Alterations in gut microbiota (GM) diversity and colonization of rederived B6 IL-10^−/−^ and C3H IL-10^−/−^ mice following disease onset**. Shannon estimate of microbial diversity plotted by Tukey box and whisker graph of B6 **(A)** and C3H **(B)** IL-10^−/−^ rederived mice (*n* = 15–25 mice per group). Statistical significance determined by one-way ANOVA with Student Newman-Keuls *post-hoc* test. *p* ≤ 0.05 is significant. Statistical significance between groups annotated by same lower case letters above box plots. Unweighted principal component analysis (PCA) of B6 **(C)** and C3H **(D)** IL-10^−/−^ rederived mice (*n* = 15–25 mice per group). Blue circles, GMCRL; green circles, GMJAX; and red circles, GMTAC. Statistical significance determined by one-way PERMANOVA (shown in Table 1).

To evaluate the contribution of the GM in the differential disease severity observed between mice, cecal contents were characterized via sequencing of the V4 hypervariable region of the 16S rRNA gene. Decreases in microbial diversity have been associated with susceptibility to inflammation and may be indicative of IBD disease severity (Ott et al., 2004; Manichanh et al., 2006; Ott and Schreiber, 2006; Frank et al., 2007). In the B6 IL-10^−/−^ model, mice rederived in surrogate dams with GMCRL had a statistically higher mean Shannon index, indicating a more diverse cecal microbial composition, than either the GMJAX or GMTAC groups (Figure 2A). In addition, the GMTAC group had a greater mean Shannon index than the GMJAX group. However, in the C3H IL10^−/−^ model, the GMCRL group had a lower mean Shannon index than either the GMJAX or GMTAC groups respectively (Figure 2B). As pups of either genetic background were exposed to similar maternal microbes at birth, these differences in microbial diversity following disease induction suggest that there is an interaction between host genetic background and GM profile in shaping microbial diversity.

To evaluate β-diversity among rederivation groups, principal component analysis (PCA) was performed. In PCA, samples that are similar in microbial composition cluster together. In the B6 IL-10^−/−^ model, PCA demonstrated that mice harboring GMCRL and GMJAX clustered independently, indicating that these are distinct microbial populations (Figure 2C, Table 1). In addition, these two groups demonstrate tight within group clustering indicating that animals within individual groups were highly similar. In contrast, the GMTAC group demonstrated greater inter- and intra- group variability along PC1 and PC2 as compared to GMCRL and GMJAX groups, indicating a less uniform microbial composition in those mice. PCAs were also examined in the context of lesion scores and no differences in clustering patterns of high or low lesions scores were noted within any of the GM profile groups (data not shown). In the C3H IL-10^−/−^ model, independent clustering of GMCRL was noted while GMTAC and GMJAX clustered closely together indicating greater compositional similarity between the latter two groups (Figure 2D, Table 1). The GMCRL and GMTAC groups demonstrated tight intra-group clustering along PC2, with GMJAX mice having increased intra-group variability along PC2.

**Table 1:** **PERMANOVA analysis of Bray-Curtis dissimilarities for bacterial OTU community structure in relation to rederivation GM group**.

**Mouse model**	**GMJAX relative to GMCRL**		**GMJAX relative to GMTAC**		**GMTAC relative to GMCRL**	
	***p*****-value**	***F*****-value**	***p*****-value**	***F*****-value**	***p*****-value**	***F*****-value**
B6 IL-10^−/−^	**0.0001**	7.076	**0.0182**	8.466	**0.0017**	10.517
C3H IL-10^−/−^	**0.0188**	9.97	**0.0176**	6.989	**0.0001**	8.586
B6 IL-10^−/−^ (CMTR)	**0.001**	9.735	**0.0299**	7.728	**0.001**	7.329

*p-values are based on 9,999 permutations with the lowest possible p-value of 0.0001. Bold face values indicate statistical significance (p ≤ 0.05)*.

### Differences in phylum and OTU relative abundance between GM profiles

**Figure 3:**
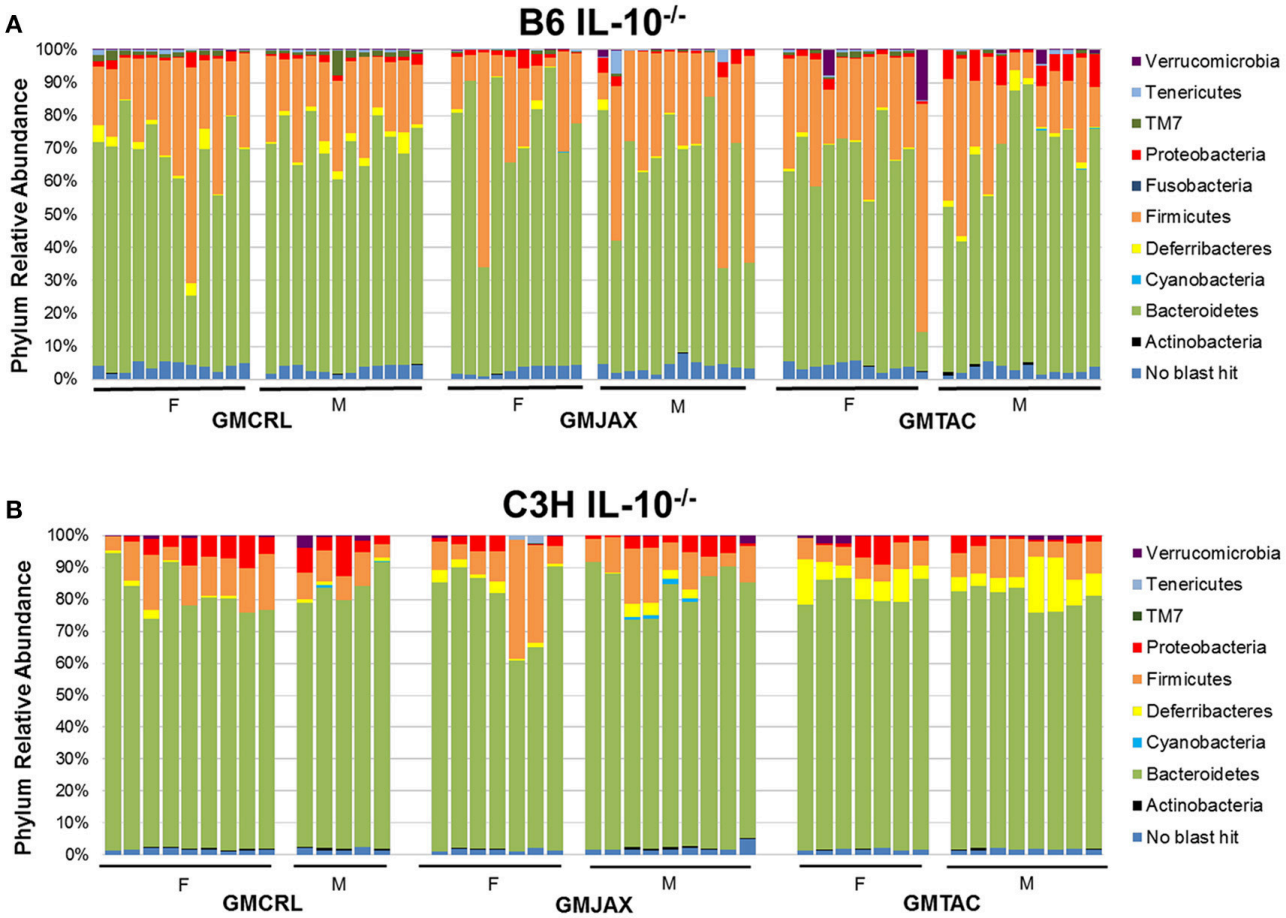
**Relative abundance of rederived groups demonstrating differences in bacterial colonization at taxonomic level of phylum**. B6 **(A)** and C3H **(B)** IL-10^−/−^ mice (*n* = 15–25 mice per group). Legend of each phyla group is shown at the right.

To identify specific microbial taxa that correlate with resistance or susceptibility to disease, relative abundance of the microbiota at both the phylum and OTU level was compared between GM profiles. In the B6 IL-10^−/−^ model, the phyla *Deferribacteres* and TM7 were enriched in mice of the GMCRL group as compared to mice in GMJAX and GMTAC groups (Figure 3A, Table S1). In the GMJAX and GMTAC groups, bacteria in the phylum *Proteobacteria* were present in greater relative abundance in mice rederived in GMTAC surrogate dams. Conversely, in the C3H IL-10^−/−^ model, a greater relative abundance of the phylum *Proteobacteria* was detected in mice of the GMCRL group (Figure 3B, Table S1) as compared to mice in the GMJAX and GMTAC groups. Moreover, the phylum *Deferribacteres* was detected at greater relative abundance in the GMTAC group as compared to GMCRL and GMJAX groups.

**Figure 4:**
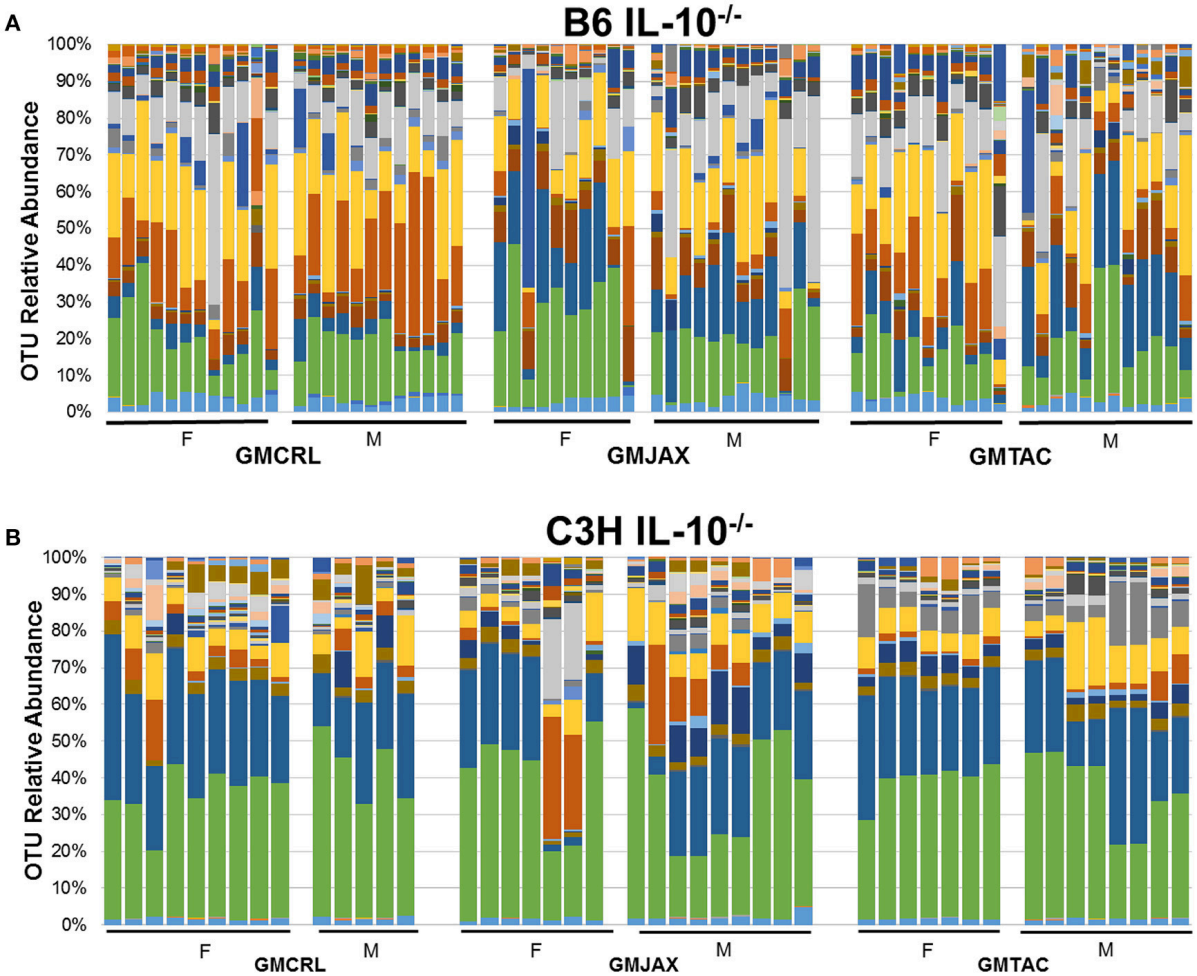
**Relative abundance of rederived groups demonstrating differences in bacterial colonization at taxonomic level of operational taxonomic unit (OTU)**. B6 **(A)** and C3H **(B)** IL-10^−/−^ mice (*n* = 15–25 mice per group). Legend of each OTU shown in Figure S5.

Resolved to the level of OTU, differences were detected in the relative abundance of several OTUs detected in all GM profiles (Figure 4A, Figure S5A, Table 2). In the B6 IL10^−/−^ model, several differences were found between GMCRL and the GMJAX and GMTAC groups; specifically, greater relative abundances of family *Rikenellaceae*, order *Bacteroidales, Mucispirillum schaedleri*, family *Clostridiaceae*, family *Ruminococcaceae*, order RF39, and family F16 were detected in mice colonized with the GMCRL profile (Table 2). In contrast, mice with the GMJAX profile had a higher relative abundance of *Bacteroides acidifaciens* and genus *Clostridium*. Similarly, in the C3H IL10^−/−^ model, the GMCRL profile had significantly higher levels of family *Mogibacteriaceae*, family *Christensenellaceae*, family *Clostridiaceae*, genus *Sutterella* relative to the other GM profiles (Figure 4B, Figure S5B, Table 3). In contrast, the GMJAX group had higher relative abundance of family *Ruminococcaceae* than either the GMCRL or GMTAC groups. Additionally, the GMTAC group both had higher relative abundance of *Mucispirillum schaedleri*, relative to GMJAX. Interestingly, the observed differences in the relative abundance of OTUs between B6 and C3H strains suggests a differential effect on colonization, as embryos from both strains were rederived on the same day using the same pools of surrogate dams.

**Table 2:** **Statistical comparison of the relative abundance of OTUs between rederivation groups within the B6 IL-10^−/−^ model**.

**Significant OTUs in B6 IL-10**^**−/−**^ **model**		**GMJAX relative to GMCRL**		**GMJAX relative to GMTAC**		**GMTAC relative to GMCRL**		**GMCRL**	**GMJAX**	**GMTAC**
**Phylum**	**OTU**	***p-*****value**	**Mean fold-change**	***p-*****value**	**Mean fold-change**	***p-*****value**	**Mean fold-change**	**Mean relative abundance (±SEM)**	**Mean relative abundance (±SEM)**	**Mean relative abundance (±SEM)**
*Bacteroidetes*	*Bacteroides acidifaciens*	<**0.001**	2.824	0.415	1.121	<**0.001**	2.523	0.053 ± 0.009	0.149 ± 0.021	0.0002 ± 1.0E−05
*Bacteroidetes*	family *Rikenellaceae*	<**0.001**	0.224	0.067	0.439	**0.002**	0.510	0.165 ± 0.0219	0.037 ± 0.014	0.091 ± 0.012
*Bacteroidetes*	order *Bacteroidales*	0.127	0.455	0.165	9.056	**0.008**	0.050	0.004 ± 0.0009	0.002 ± 0.0001	0.0002 ± 0.0001
*Deferribacteres*	*Mucispirillum schaedleri*	<**0.001**	0.179	0.134	0.356	**0.010**	0.502	0.021 ± 0.003	0.004 ± 0.001	0.0105 ± 0.003
*Firmicutes*	family *Clostridiaceae*	<**0.001**	0.224	0.506	2.336	<**0.001**	0.096	0.009 ± 0.0001	0.002 ± 6.1E−05	8.3E−05 ± 2.2E−05
*Firmicutes*	family *Peptococcaceae*	**0.003**	1.527	^*^	0.000	0.310	1.288	0.0002 ± 2.71E−05	0.000 ± 0.000	0.0002 ± 5.2E−05
*Firmicutes*	family *Peptostreptococcaceae*	**0.007**	0.198	0.624	3.07	**0.003**	0.0644	0.0012 ± 0.0004	0.002 ± 9.5E−05	7.9E−05 ± 6.5E−05
*Firmicutes*	family *Ruminococcaceae*	<**0.001**	0.473	**0.002**	0.503	0.649	0.941	0.021 ± 0.001	0.001 ± 0.002	0.019 ± 0.003
*Firmicutes*	genus *Allobaculum*	0.966	0.790	0.017	0.073	**0.004**	10.778	0.0004 ± 0.0001	0.003 ± 0.0002	0.004 ± 0.001
*Firmicutes*	genus *Clostridium*	0.238	1.474	**0.009**	20.55	0.044	0.096	0.0007 ± 0.0002	0.001 ± 0.0003	5.1E−05 ± 2.4E−05
*Proteobacteria*	genus *Bilophila*	^*^	0.000	<**0.001**	0.0002	<**0.001**	0.000	0.000 ± 0.000	1.0E−05 ± 1.0E−06	0.007 ± 0.002
*Proteobacteria*	genus *Sutterella*	0.446	0.399	**0.007**	0.137	0.013	2.916	0.006 ± 0.001	0.002 ± 0.0008	0.016 ± 0.005
*Tenericutes*	order RF39	<**0.001**	0.036	**0.005**	0.066	**0.002**	0.547	0.005 ± 0.0007	0.0002 ± 6.9E−05	0.003 ± 0.0006
TM7	family F16	<**0.001**	0.197	0.302	0.474	**0.005**	0.415	0.0137 ± 0.002	0.003 ± 0.0008	0.006 ± 0.001

* *Statistical comparison not applicable due to 0.000 OTU relative abundance*.

*p-value, mean fold change, and mean relative abundance are shown. Statistical significance and interactions of GM profile, sex, and OTU were determined using two-way ANOVA with Student Newman-Keuls post-test. Bold face values indicate statistical significance (p ≤ 0.01)*.

**Table 3:** **Statistical comparison of the relative abundance of OTUs between rederivation groups within the C3H IL-10^−/−^ model**.

**Significant OTUs in C3H IL-10**^**−/−**^ **model**		**GMJAX relative to GMCRL**		**GMJAX relative to GMTAC**		**GMTAC relative to GMCRL**		**GMCRL**	**GMJAX**	**GMTAC**
**Phylum**	**OTU**	***p-*****value**	**Mean fold change**	***p-*****value**	**Mean fold change**	***p-*****value**	**Mean fold change**	**Mean relative abundance (±SEM)**	**Mean relative abundance (±SEM)**	**Mean relative abundance (±SEM)**
*Deferribacteres*	*Mucispirillum schaedleri*	0.384	2.235	<**0.001**	0.225	<**0.001**	9.338	0.0078 ± 0.002	0.017 ± 0.004	0.078 ± 0.012
*Firmicutes*	family *Mogibacteriaceae*	<**0.001**	0.099	0.671	2.715	<**0.001**	0.038	0.011 ± 0.002	0.001 ± 0.0004	0.0004 ± 6.6*E*−06
*Firmicutes*	family *Christensenellaceae*	0.020	0.312	0.330	4.832	**0.006**	0.064	0.003 ± 0.001	0.001 ± 0.0004	0.0002 ± 8.9E−05
*Firmicutes*	family *Clostridiaceae*	<**0.001**	0.242	0.396	3.082	<**0.001**	0.076	0.003 ± 0.002	7.0E−05 ± 3.0E−05	2.2E−05 ± 5.0E−06
*Firmicutes*	family *Ruminococcaceae*	0.162	1.627	**0.006**	3.891	0.113	0.430	0.006 ± 0.001	0.010 ± 0.002	0.003±.0004
*Proteobacteria*	genus *Sutterella*	**0.004**	0.382	0.448	1.678	**0.002**	0.243	0.039 ± 0.008	0.015 ± 0.004	0.009 ± 0.002

*p-value, mean fold change, and mean relative abundance are shown. Statistical significance and interactions of GM profile, sex, and OTU were determined using two-way ANOVA with Student Newman-Keuls post-test. Bold face values indicate statistical significance (p ≤ 0.01)*.

While differential disease susceptibility may be a function of differential abundance of specific taxa, it may also be explained by the presence or absence of select taxa unique to each GM profile. Interestingly, in B6 IL10^−/−^ mice we found several unique OTUs including *Alistipes massiliensis, Roseburia faecis, Clostridium saccharogumia*, and genus *Megamonas* were found in mice rederived in GMCRL dams, whereas *Eubacterium dolichum* was detected only in the GMTAC rederived group. *Aggregatibacter pneumotropica* was only found in GMJAX and GMTAC groups with *Propionibacterium acnes* and genus *Phascolarctobacterium* detected in only GMCRL and GMJAX groups; Candidatus *Arthromitus* (segmented filamentous bacterium), genus *Anaerotruncus* and *Oxalobacter formigenes* were detected in both the GMTAC and GMCRL groups.

In contrast, in the C3H IL10^−/−^ mice, the GMCRL group harbored *Prevotella copri, Alistipes massiliensis*, family *Paraprevotellaceae, Enterococcus casseliflavus, Faecalibacterium prausnitzii*, genus *Phascolarctobacterium*, genus *Fusobacterium*, genus *Anaerobiospirillum*, genus *Prevotella*, and genus *Anaerotruncus*. *Parabacteroides distasonis*, family *Porphyromonadaceae*, genus *Enterococcus*, genus *Roseburia*, family *Desulfovibrionaceae*, and *Flexispira rappini* were found to overlap between the GMJAX and GMTAC groups; genus *Turicibacter* and family *Peptostreptococcaceae* were detected in both the GMJAX and GMCRL groups; genus *Anaerotruncus* and Candidatus *Arthromitus* were specific to the GMTAC and GMCRL groups. Differences in the presence of these OTUs and their interaction with other OTUs present at significantly different levels may account for observed differences in disease severity.

### GM profile affects *Helicobacter hepaticus* colonization

**Figure 5:**
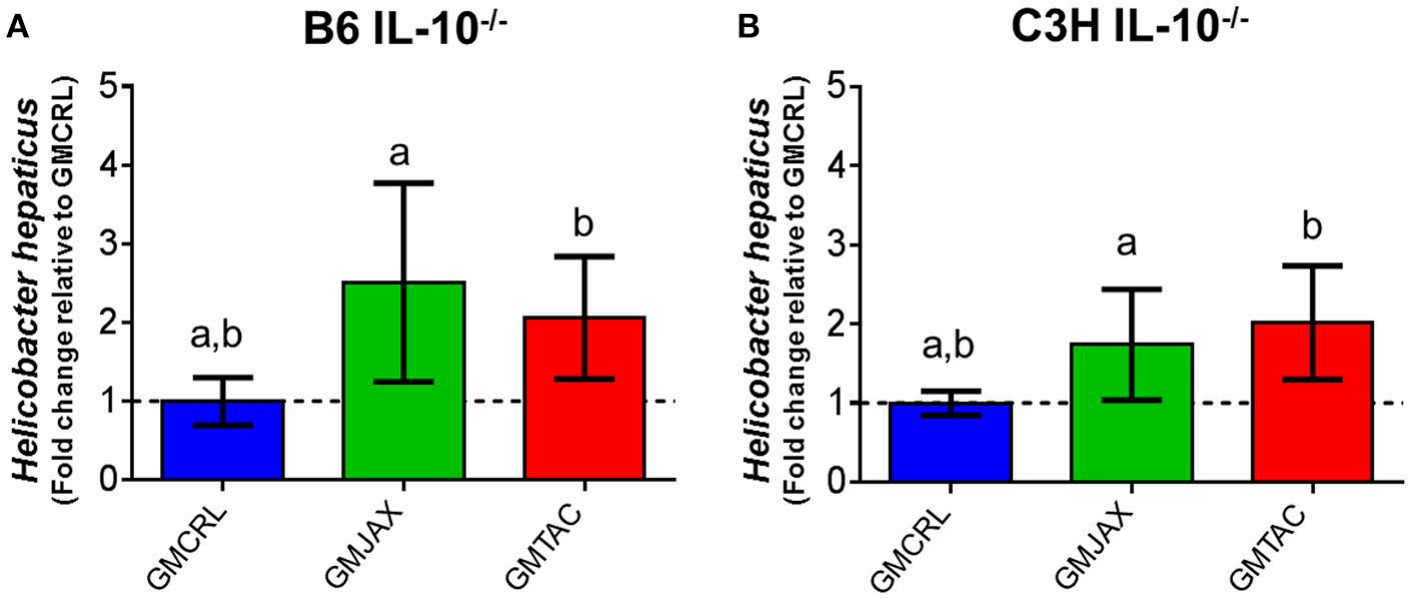
**Relative abundance of *Helicobacter hepaticus* within rederived groups in B6 IL-10^−/−^ and C3H IL-10^−/−^ mice**. B6 **(A)** and C3H **(B)** IL-10^−/−^ rederived mice (*n* = 15–25 mice per group). Statistical significance determined by one-way ANOVA with Student Newman-Keuls post-test. Bars represent SEM. *p* ≤ 0.05 is significant. Statistical significance between groups annotated by same lower case letters above bar charts.

*Helicobacter spp*. are thought to be a provocateur of intestinal inflammation and dysbiosis (Fox, 2007; Jergens et al., 2007) and as such are necessary for the induction of disease in the IL-10^−/−^ model. To determine if the observed differences in lesion score among GM groups could be due to differences in *H. hepaticus* colonization, we performed semi-quantitative real time PCR on cecal bacterial contents. In both B6 IL-10^−/−^ (Figure 5A) and C3H IL-10^−/−^ (Figure 5B) mice, mice colonized with GMCRL had significantly lower relative abundance of *H. hepaticus* than mic colonized with GMJAX or GMTAC. All mice received the same *H. hepaticus* inoculum post-weaning and are genetically identical, suggesting differences between GM profiles resulted in differing colonization resistance against *H. hepaticus*.

### Alterations in disease severity and GM composition independent of surrogate dam genetics and maternal care

**Figure 6:**
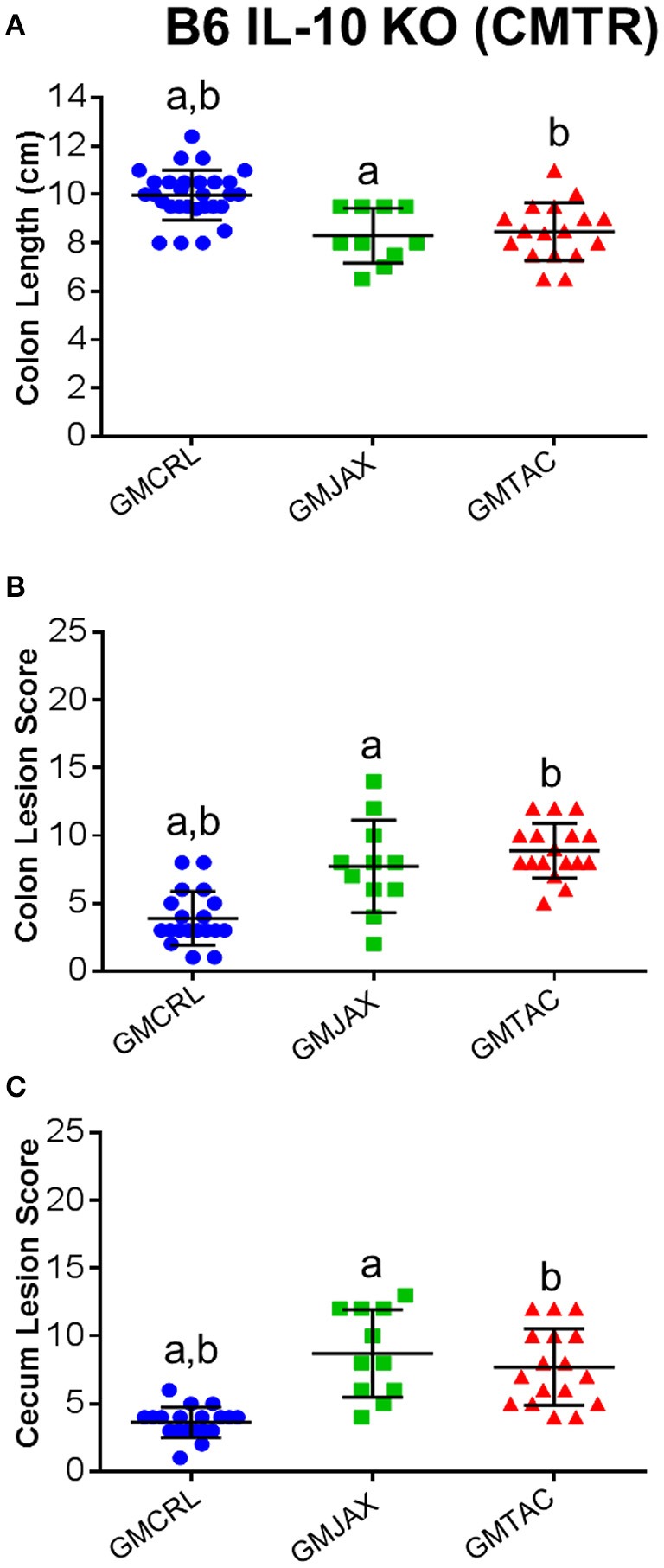
**Colonic and cecal disease is decreased in B6 IL-10^−/−^ mice following CMTR to CD1_GMCRL_ surrogate dams**. Colon length **(A)**, colon lesion score **(B)**, and cecal lesion score **(C)** of B6 IL-10^−/−^ mice with different gut microbiota (GM). Statistical significance determined by one-way ANOVA with Student Newman-Keuls post-test. Bars indicate mean and SEM. *p* ≤ 0.05 is significant. Statistical significance between groups annotated by same lower case letters above dot plots.

Because the initial complex microbiota targeted rederivation (CMTR) was performed using surrogate dams with varying genetic backgrounds, it is possible that differences in maternal care, *in utero* environment, or epigenetic factors contributed to the observed differential disease severity. To address this consideration, we established breeding colonies of CD1 dams harboring either the GMCRL, GMJAX, or GMTAC profiles. To do so, CMTR was used to transfer CD1 embryos to three surrogate dams (Crl:CD1, C57BL/6J, and C57BL/6NTac), and thus generate pups with the previously studied GM profiles (Figure S2). Offspring were then used to establish three separate breeding colonies using an outbred mating scheme. Colonies were monitored for two generations using next generation sequencing to confirm expected differences in GM composition and complexity. PCA of first and second generation females at 8–10 weeks of age confirmed three distinct GM profiles (*p* ≤ 0.001; *F* = 12.19; Figure S3). Second generation females were used as surrogate dams for CMTR of the B6 IL-10^−/−^ model (Figure S4). As in the previous studies, pups were inoculated with *H. hepaticus* at 24 and 26 days of age and disease severity was assessed at 111 days of age. Grossly, B6 IL-10^−/−^ pups harboring GMCRL had a significantly greater colon length than the GMJAX or GMTAC groups (Figure 6A). In addition, GMCRL-colonized pups had lower cecal and colonic histopathologic lesion scores compared to GMJAX and GMTAC groups, in agreement with findings from studies using inbred surrogate dams (Figures 6B,C).

**Figure 7:**
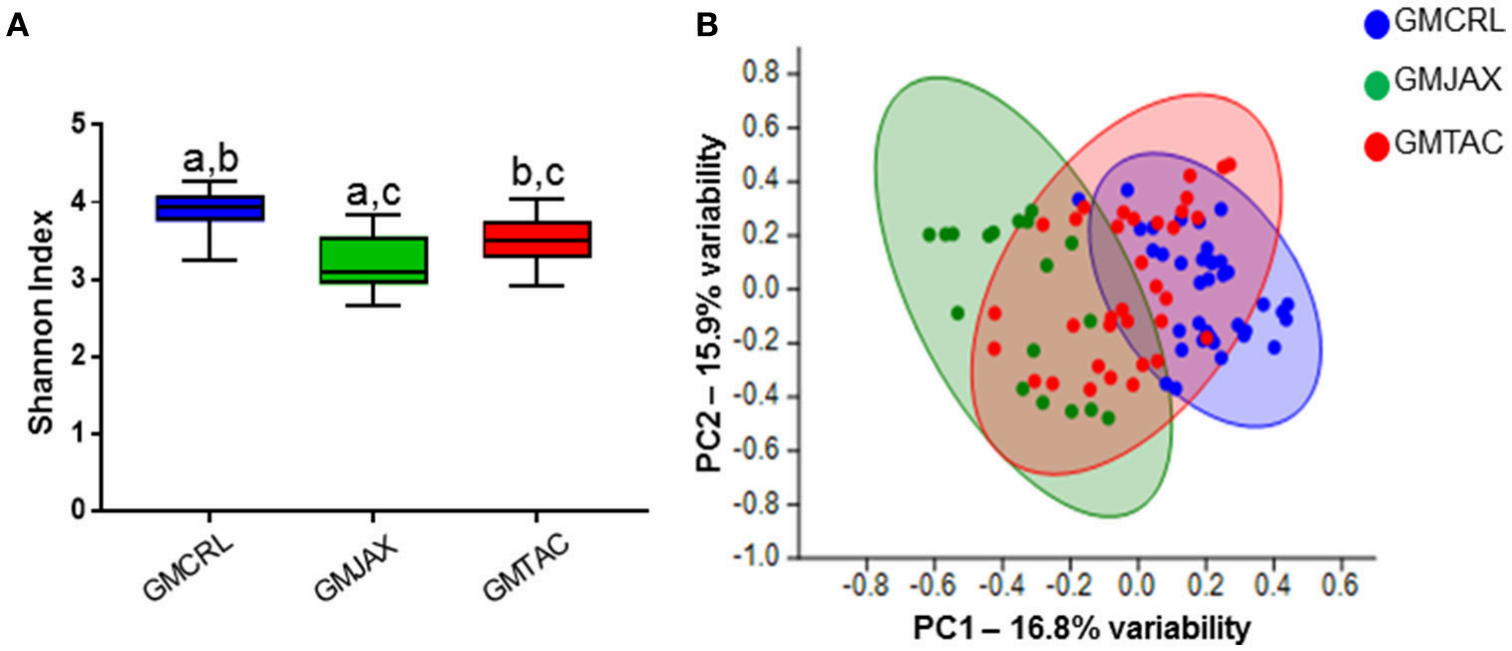
**Alterations in gut microbiota (GM) diversity and colonization in B6 IL-10^−/−^ mice following CMTR to CD1 surrogate dams**. **(A)** Shannon estimate of microbial diversity plotted by Tukey box and whisker graph of B6 IL-10^−/−^ rederived mice (*n* = 15–25 mice per group). Statistical significance determined by one-way ANOVA with Student Newman-Keuls post-test. *p* ≤ 0.05 is significant. Statistical significance between groups annotated by same lower case letters above bar charts. **(B)** Unweighted principal component analysis (PCA) of B6 IL-10^−/−^ rederived mice (*n* = 15–25 mice per group). Blue circles, GMCRL; green circles, GMJAX; and red circles, GMTAC. Statistical significance determined by one-way PERMANOVA (shown in Table 1).

**Figure 8:**
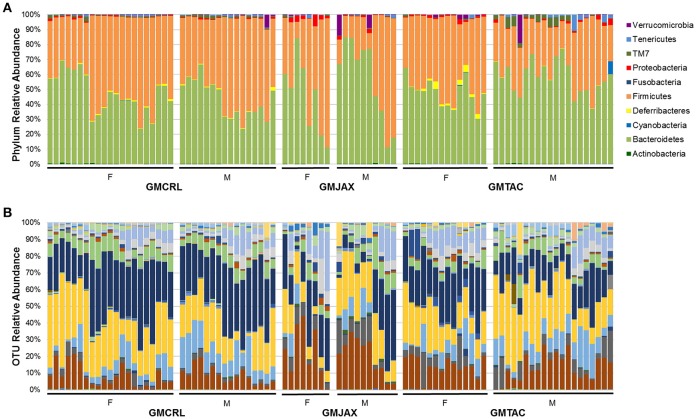
**Relative abundance of B6 IL-10^−/−^ mice following CMTR to CD1 surrogate dams at taxonomic level of phylum and operational taxonomic unit (OTU). (A)** Bar charts of relative abundance of phyla. Legend of phyla shown at right. **(B)** Bar charts of relative abundance of OTU. *n* = 20–35 per group. Legend of each OTU shown in Figure S5.

Again confirming the previous findings in the B6 IL-10^−/−^ model, sequencing of cecal contents revealed that the GMCRL group had increased diversity when compared to the GMJAX and GMTAC groups (Figure 7A). Moreover, the GMTAC group was more diverse than the GMJAX group. PCA demonstrated that each group maintained three distinct microbial profiles (Figure 7B, Table 1). To further investigate GM composition, relative abundance at the phylum and OTU level were examined. As in our initial experiments using the B6 IL-10^−/−^ model, there was increased relative abundance of phylum *Proteobacteria* in both the GMJAX and GMTAC groups (Figure 8A, Table S1). In contrast to our previous findings, subjectively there was lower relative abundance of *Deferribacteres* in GMJAXmice, with increased relative abundance observed in GMTAC males. At the OTU level, we found several OTUs which differed between groups and had similar relative abundance patterns as seen in the previous study including *B. acidifaciens*, family *Rikenellaceae*, order *Bacteroidales, Mucispirillum schaedleri*, family *Clostridiaceae*, genus *Clostridium*, and family F16 (Figure 8B, Figure S5, Table 4). In addition, we found several species within the phyla *Bacteroidetes, Firmicutes*, and *Proteobacteria* that differed between groups including genus *Bacteroides*, family *Mogibacteriaceae*, family *Christensenellaceae*, genus rc4-4, order *Clostridiales*, family *Enterobacteriaceae*, and genus *Helicobacter*.

**Table 4:** **Statistical comparison of the relative abundance of OTUs between CMTR groups within the B6 IL-10^−/−^ model**.

**Significant OTUs in B6 IL-10**^**−/−**^ **(CMTR to CD1 surrogates)**		**GMJAX relative to GMCRL**		**GMJAX relative to GMTAC**		**GMTAC relative to GMCRL**		**GMCRL**	**GMJAX**	**GMTAC**
**Phylum**	**OTU**	***p-*****value**	**Mean fold change**	***p-*****value**	**Mean fold change**	***p-*****value**	**Mean fold change**	**Mean relative abundance (±SEM)**	**Mean relative abundance (±SEM)**	**Mean relative abundance (±SEM)**
*Bacteroidetes*	*Bacteroides acidifaciens*	**0.002**	3.260	0.620	1.16	<**0.001**	2.809	0.022 ± 0.003	0.072 ± 0.013	0.062 ± 0.01
*Bacteroidetes*	family *Rikenellaceae*	**0.006**	0.458	**0.003**	0.446	0.979	1.027	0.095 ± 0.01	0.043 ± 0.014	0.100 ± 0.01
*Bacteroidetes*	genus *Bacteroides*	<**0.001**	2.527	0.036	1.554	**0.005**	1.625	0.0834 ± 0.009	0.211 ± 0.306	0.136 ± 0.012
*Bacteroidetes*	order *Bacteroidales*	**0.01**	0.000	0.058	0.000	0.595	0.387	0.0002 ± 4.12E−05	0.000 ± 0.000	0.0001 ± 2.3E−05
*Deferribacteres*	*Mucispirillum schaedleri*	0.016	0.0005	<**0.001**	0.0004	0.012	1.592	0.005 ± 0.0007	3.36*E*−06 ± 1.8*E*−06	0.008 ± 0.002
*Firmicutes*	family *Mogibacteriaceae*	<**0.001**	0.374	0.032	0.564	**0.005**	0.662	0.0008 ± 0.00005	0.0003 ± 6.83E−05	0.0005 ± 6.9E−05
*Firmicutes*	family *Christensenellaceae*	<**0.001**	2.858	<**0.001**	2.332	0.691	1.226	0.0003 ± 0.00004	0.001 ± 0.0001	0.0003 ± 4.5E−05
*Firmicutes*	family *Clostridiaceae*	**0.01**	0.288	0.916	0.977	**0.005**	0.294	0.0001 ± 0.00024	0.0003 ± 6.04E−05	0.0003 ± 7.6E−05
*Firmicutes*	genus *Clostridium*	**0.01**	0.000	0.688	0.000	**0.004**	0.432	0.0005 ± 0.0001	0.000 ± 0.000	7.6E−05 ± 4.2E−06
*Firmicutes*	genus rc4-4	<**0.001**	0.035	0.044	0.088	<**0.001**	0.402	0.0028 ± 0.0003	9.74E−05 ± 1.02E−06	0.001±.0002
*Firmicutes*	order *Clostridiales*	<**0.001**	0.611	0.776	0.960	<**0.001**	0.636	0.318 ± 0.015	0.19 ± 0.035	0.202 ± 0.015
*Proteobacteria*	family *Enterobacteriaceae*	**0.002**	21.629	**0.006**	24.616	0.974	0.879	8.93E−06 ± 3.3E−07	0.002 ± 0001	7.84E−06 ± 3.0E−06
*Proteobacteria*	genus *Helicobacter*	<**0.001**	5.346	<**0.001**	2.301	0.095	2.323	0.003 ± 0.0005	0.025 ± 0.005	0.007 ± 0.001
TM7	family F16	0.108	0.294	**0.008**	0.163	0.075	1.801	0.009 ± 0.001	0.002 ± 0.0008	0.016 ± 0.003

*P-value, mean fold change, and mean relative abundance are shown. Statistical significance and interactions of GM profile, sex, and OTU were determined using two-way ANOVA with Student Newman-Keuls post-test. Bold face values indicate statistical significance (p ≤ 0.01)*.

**Figure 9:**
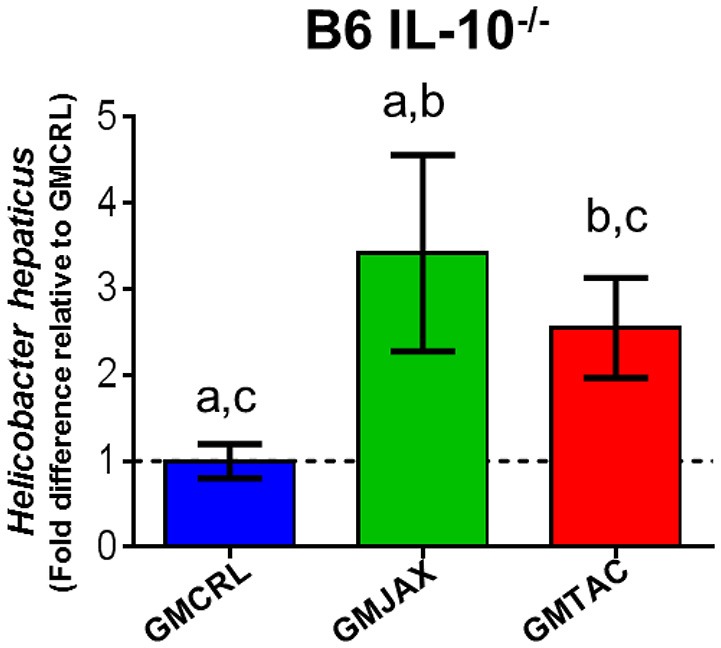
**Relative abundance of *Helicobacter hepaticus* colonization of B6 IL-10^−/−^ mice following CMTR to CD1 surrogate dams**. Statistical significance determined by one-way ANOVA with Student Newman-Keuls post-test. *p* ≤ 0.05 is significant. Statistical significance between groups annotated by same lower case letters above bar charts. *n* = 20–35 per group.

To determine if the observed differences in lesion score among GM groups could be explained by differences in *H. hepaticus* colonization, we performed semi-quantitative real time PCR on cecal contents. As observed in our previous experiments, we found that B6 IL-10^−/−^ mice with GMCRL were colonized with *H. hepaticus* to a lesser degree than mice colonized with either GMJAX or GMTAC profiles (Figure 9).

## Discussion

With increasing availability of culture-independent technologies, there are abundant studies providing evidence of the importance of the GM in both GI and systemic disease. However, research using human subjects remains confounded by genetic and environmental variables. The use of mouse models allows longitudinal studies to characterize the GM with careful control of both genetic and environmental variables. Manipulation of individual microbes, or use of GF mice, while useful in certain contexts, is somewhat reductionist and does not take into account the interactions between naturally occurring complex GI populations.

In this study, we demonstrate that complex microbiota targeted rederivation (CMTR) by embryo transfer can be used as a method to study the influence of complex microbial communities in rodent models of disease. We surgically implanted genetically identical embryos, collected from IL-10^−/−^ mice, into surrogate dams harboring different GM varying in composition and complexity to produce IL-10^−/−^ pups colonized with the desired maternal GM profiles. Using these methods, we show that the GM of surrogate dam greatly influences colonic and cecal disease phenotype in both the B6 IL-10^−/−^ and C3H IL-10^−/−^ mouse models. In both genetic backgrounds, IL-10^−/−^ mice born to surrogate dams colonized with GMCRL had longer colon length and lower mean colonic and cecal lesion scores indicating a lower disease phenotype in these animals. In addition, we found that on the B6 background, mice born to GMTAC-colonized surrogate dams had the most severe disease phenotype while mice rederived to GMJAX-colonized surrogate dams had intermediate disease severity. On the C3H background, IL-10^−/−^ mice born to surrogate dams harboring either GMJAX or GMTAC had significantly higher colon and cecal lesion scores relative to mice born to dams colonized with GMCRL. Interestingly, we found that in both IL-10^−/−^ strains tested, the GMCRL group had the lowest relative abundance of *H. hepaticus* in cecal samples. This suggests that the composition of the bacterial species present in the GMCRL profile may be interfering with colonization by this critical provocateur of disease.

Previous IBD studies have suggested that decreased GI microbial diversity correlates with increased inflammation and disease severity (Ott et al., 2004; Manichanh et al., 2006; Frank et al., 2007). We show that differences in microbial diversity were present between GM groups dependent on surrogate dam used for rederivation and the host genetic background. In the B6 IL-10^−/−^ model, we found increased microbial diversity in the GMCRL group which had the lowest disease score. In contrast, C3H IL-10^−/−^ mice had a decreased diversity in the GMCRL group. This unexpected interaction between genetic background and GM profile may be a reflection of an abnormal innate immune response, as C3H IL-10^−/−^ mice carry a mutation in the TLR4 gene rendering innate immune cells such as macrophages unable to respond to LPS (Watson et al., 1978; Poltorak et al., 1998).

In both genetic backgrounds tested, we found differences in several physiological bacteria associated with the strain and vendor of the surrogate dams chosen for rederivation which likely contribute to the differences in observed disease severity between groups. In the B6 background, genus *Clostridium*, family *Ruminococcaceae*, and family *Rikenellaceae* were present at significantly lower relative abundance in the GMJAX- and GMTAC-colonized mice, the groups that developed more severe inflammation. In addition, in both genetic backgrounds, we detected lower relative abundance of family *Clostridiaceae*. These data are consistent with previous findings in human patients with confirmed IBD (Frank et al., 2007; Joossens et al., 2011; Sartor and Mazmanian, 2012; Walters et al., 2014) suggesting that increased relative abundance of these taxa may contribute to the protection from disease observed in the GMCRL group. In addition, we found that, in both B6 and C3H IL-10^−/−^ mice, there was a greater relative abundance of phylum *Proteobacteria* in the GMJAX and GMTAC profiles, and greater relative abundance of genus *Sutterella*, genus *Bilophila*, and family *Enterobacteriaceae* in the B6 IL-10^−/−^ mice and genus *Sutterella* in the C3H IL-10^−/−^ mice. Increased abundance of these OTUs has also been suggested to play a role in IBD severity in humans (Gophna et al., 2006; Baumgart et al., 2007; Frank et al., 2007).

Previous studies have suggested that normal enteric bacteria are essential for the development of chronic intestinal colitis in IL-10^−/−^ mice (Sellon et al., 1998). These mice exhibit varying degrees of disease severity dependent on housing conditions used. Specifically, when housed under germ-free conditions these mice fail to develop colitis whereas specific pathogen-free (SPF) or conventionally housed mice develop chronic colitis. When gnotobiotic IL-10^−/−^ mice are monocolonized with *H. hepaticus* they fail to develop intestinal disease, implicating the role of the normal GM in disease pathogenesis (Dieleman et al., 2000). Moreover, differential effects of institutional housing and associated variation in GM composition influences disease severity (Yang et al., 2013). Taken as a whole, these data underscore the importance of the GM in IBD pathogenesis. However, the mechanism of these bacterial interactions and their role in inducing disease severity remains to be fully explored. CMTR provides a platform to further examine these complex host:GM interactions.

With recent concerns of ensuring animal models of disease are consistent and reproducible it becomes paramount that a clear understanding of the contribution of the GM is clearly defined (Collins and Tabak, 2014; Perrin, 2014). Along with the inclusion of both sexes, randomization, and better powered studies, we believe that differences in the GM may also explain some of this poor reproducibility. CMTR allows generation of isogenic mice harboring distinct, well-characterized GM or, alternatively, could be used to generate genetically disparate colonies of mice seeded with the same GM. As such, we feel that CMTR will have a significant impact on the methods used to study the GM contribution to health and disease. While we used this technique in a model of IBD and have also used it successfully in a rat model of colorectal cancer (Ericsson et al., 2015a), it could be used in any model in which the GM is hypothesized to have an influence.

## Ethics statement

The current study was conducted in accordance with the guidelines set forth by the Guide for the Use and Care of Laboratory Animals and the Public Health Service Policy on Humane Care and Use of Laboratory Animals. All studies and protocols (#7914 and #8720) were approved by the University of Missouri Institutional Animal Care and Use Committee.

## Availability of data and material

The datasets generated and analyzed during the current study are available at the NCBI SRA database, Bioproject number PRJNA384164.

## Author contributions

Conceived and designed the experiments: MH, CF, AE. Performed the experiments: MH. Analyzed the data: MH. Contributed reagents/materials/analysis tools: CF. Wrote the paper: MH, CF, AE.

## Funding

This research was funded by grants from the National Institutes of Health (NIH U42 OD010918-13 and NIH T32 OD011126) to the University of Missouri Mutant Mouse Resource and Research Center (www.mmrrc.org) and a T32 training grant to the University of Missouri Comparative Medicine Program (cmp.missouri.edu). The funders had no role in study design, data collection and analysis, decision to publish, or preparation of the manuscript.

### Conflict of interest statement

The authors declare that the research was conducted in the absence of any commercial or financial relationships that could be construed as a potential conflict of interest.

## Supplementary material

Figure S1 **Experimental design used to generate IL-10^−/−^ pups with different GI microbiota (GM)**. Schematic diagram showing embryo transfer scheme used to generate B6.129P2-Il10^*tm*1*Cgn*^/J (B6 IL-10^−/−^) and C3Bir.129P2(B6)-Il10^*tm*1*Cgn*^/J (C3H IL-10^−/−^) pups to Crl:CD1_gmCRL_, C57BL/6J_gmJAX_, C57BL/6NTac_gmTAC_ surrogate dams. Pups were inoculated with *H. hepaticus* at 24 and 26 days of age and necropsied at 111 days of age. Cecal contents were submitted for sequencing and cecal and colonic disease evaluated.

Figure S2 **Experimental design used to generate CD1 mice with different GI microbiota (GM)**. Schematic diagram showing embryo transfer scheme use to rederived CD1 mice to Crl:CD1_GMCRL_, C57BL/6J_GMJAX_, C57BL/6NTac_GMTAC_ surrogate dams. At maturity pups were mated using an outbred mating scheme and maintained as a breeding colony for two generations. Second generation 8–10 week old females were used for subsequent complex microbiota targeted rederivation (CMTR) of B6 IL-10^−/−^ mice.

Figure S3 **Principal component analysis of the gut microbiota (GM) of rederived CD1 mice**. Representative samples from 1st and 2nd generation females at 8–10 weeks of age. *n* = 45–50. Blue circles = GMCRL; green circles = GMJAX; and red circles = GMTAC. Statistical significance determined using one-way PERMANOVA (*p* ≤ 0.05 significant).

Figure S4 **Experimental design used to generate IL-10^−/−^ pups with different GI microbiota (GM)**. Schematic diagram showing embryo transfer scheme used to generate B6.129P2-Il10^*tm*1*Cgn*^/J (B6 IL-10^−/−^) pups rederived to second generation CD1_GMCRL_, CD1_GMJAX_, CD1_GMTAC_ surrogate dams from previously established colonies. Pups were inoculated with *H. hepaticus* at 24 and 26 days of age and necropsied at 111 days of age. Cecal contents were submitted for sequencing and cecal and colonic disease evaluated.

Figure S5 **OTU relative abundance bar chart color legend. (A)** OTU legend for Figures 4A,B. **(B)** OTU legend for Figure 8B.

Table S1 **Statistical comparison of the relative abundance of phyla between rederivation groups within the B6 IL-10^−/−^ and C3H IL-10^−/−^ models**. *P*-value, mean fold change, and mean relative abundance are shown. Statistical significance and interactions of GM profile, sex, and OTU were determined using two-way ANOVA with Student Newman-Keuls post-test. Bold face values indicate statistical significance (*p* ≤ 0.01).
