# Supplementary figures and images for: Differing Complex Microbiota Alter Disease Severity of the IL-10−/− Mouse Model of Inflammatory Bowel Disease
Source: Front Microbiol. 2017 May 11;8:792. doi: 10.3389/fmicb.2017.00792 (PMC5425584; doi:10.3389/fmicb.2017.00792)

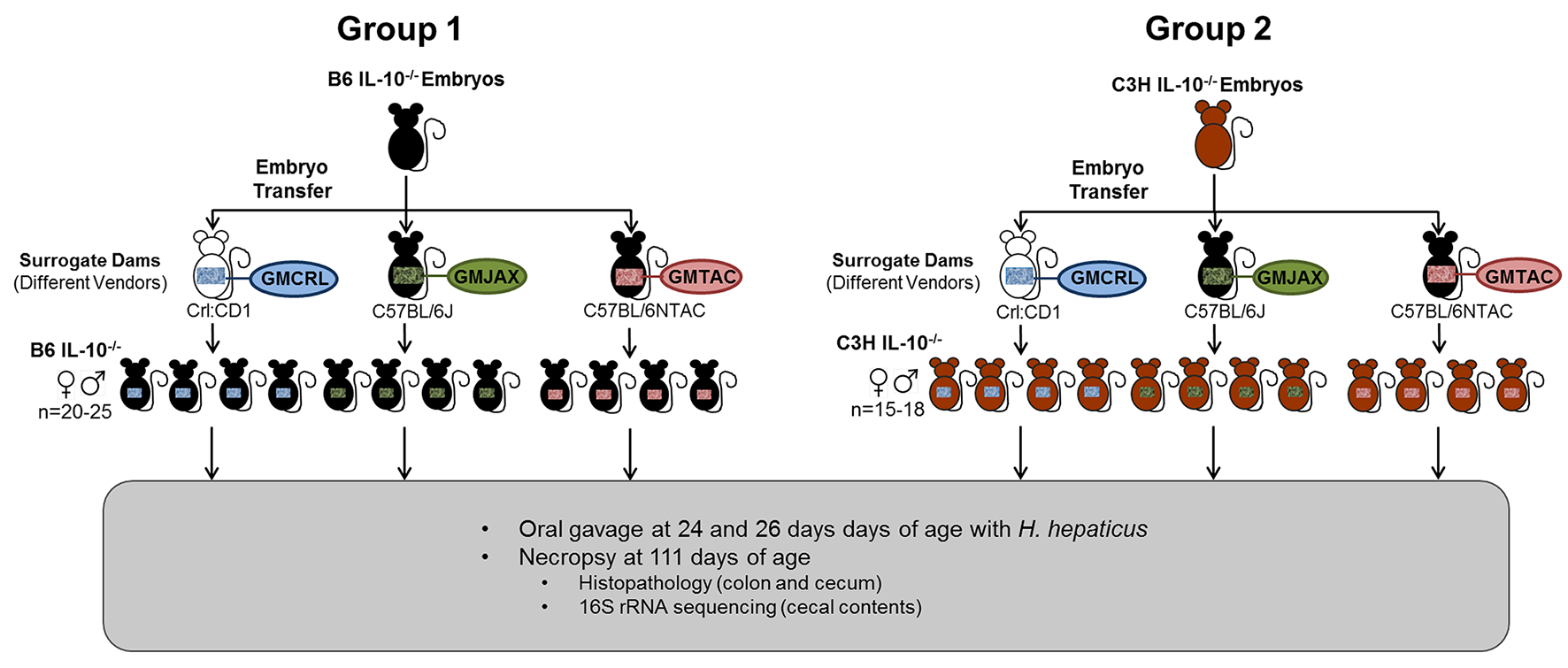

Supplement: Figure S1 — Experimental design used to generate IL-10−/− pups with different GI microbiota (GM). Schematic diagram showing embryo transfer scheme used to generate B6.129P2-Il10tm1Cgn/J (B6 IL-10−/−) and C3Bir.129P2(B6)-Il10tm1Cgn/J (C3H IL-10−/−) pups to Crl:CD1gmCRL, C57BL/6JgmJAX, C57BL/6NTacgmTAC surrogate dams. Pups were inoculated with H. hepaticus at 24 and 26 days of age and necropsied at 111 days of age. Cecal contents were submitted for sequencing and cecal and colonic disease evaluated. [file Image1.TIF]

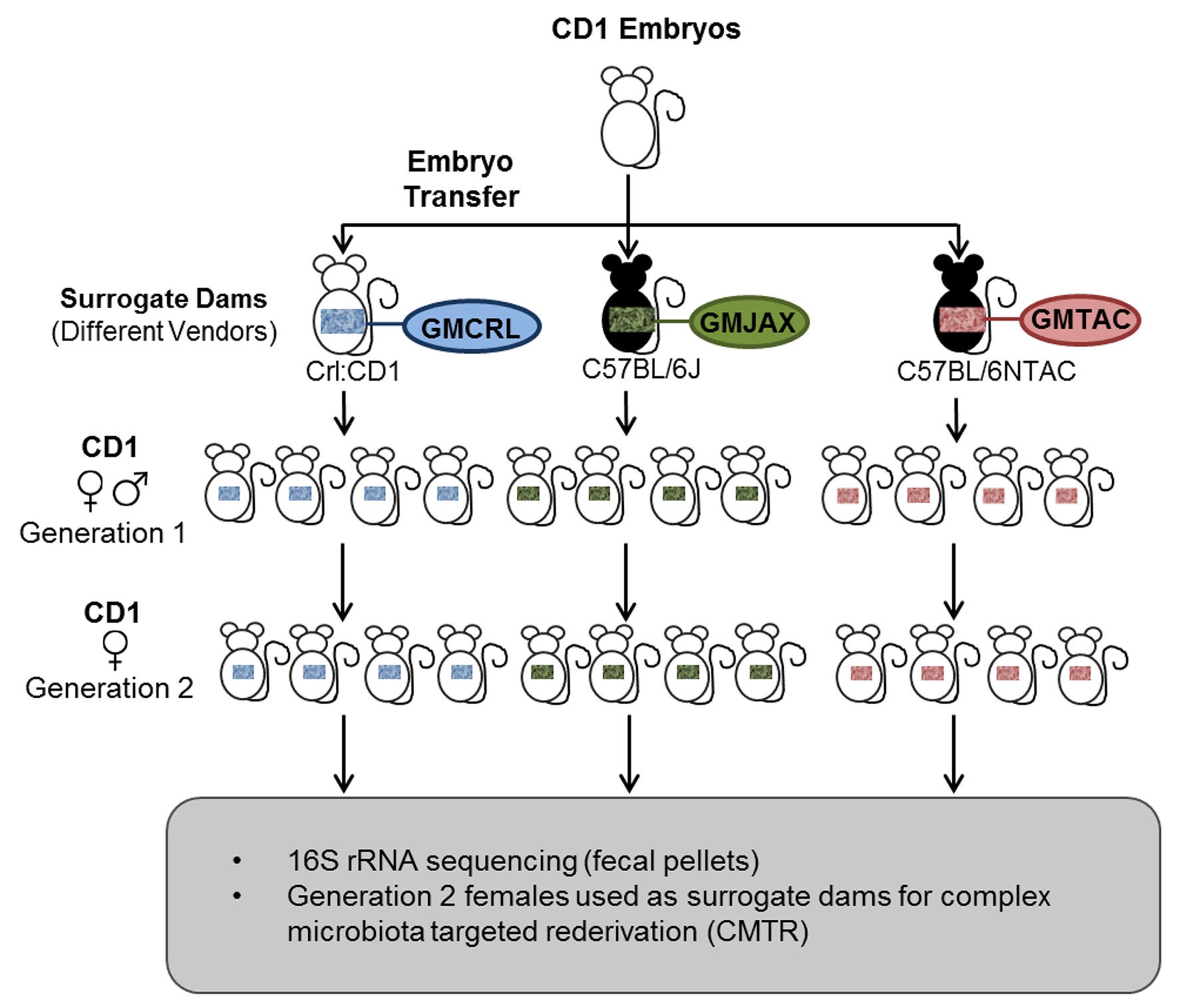

Supplement: Figure S2 — Experimental design used to generate CD1 mice with different GI microbiota (GM). Schematic diagram showing embryo transfer scheme use to rederived CD1 mice to Crl:CD1GMCRL, C57BL/6JGMJAX, C57BL/6NTacGMTAC surrogate dams. At maturity pups were mated using an outbred mating scheme and maintained as a breeding colony for two generations. Second generation 8–10 week old females were used for subsequent complex microbiota targeted rederivation (CMTR) of B6 IL-10−/− mice. [file Image2.TIF]

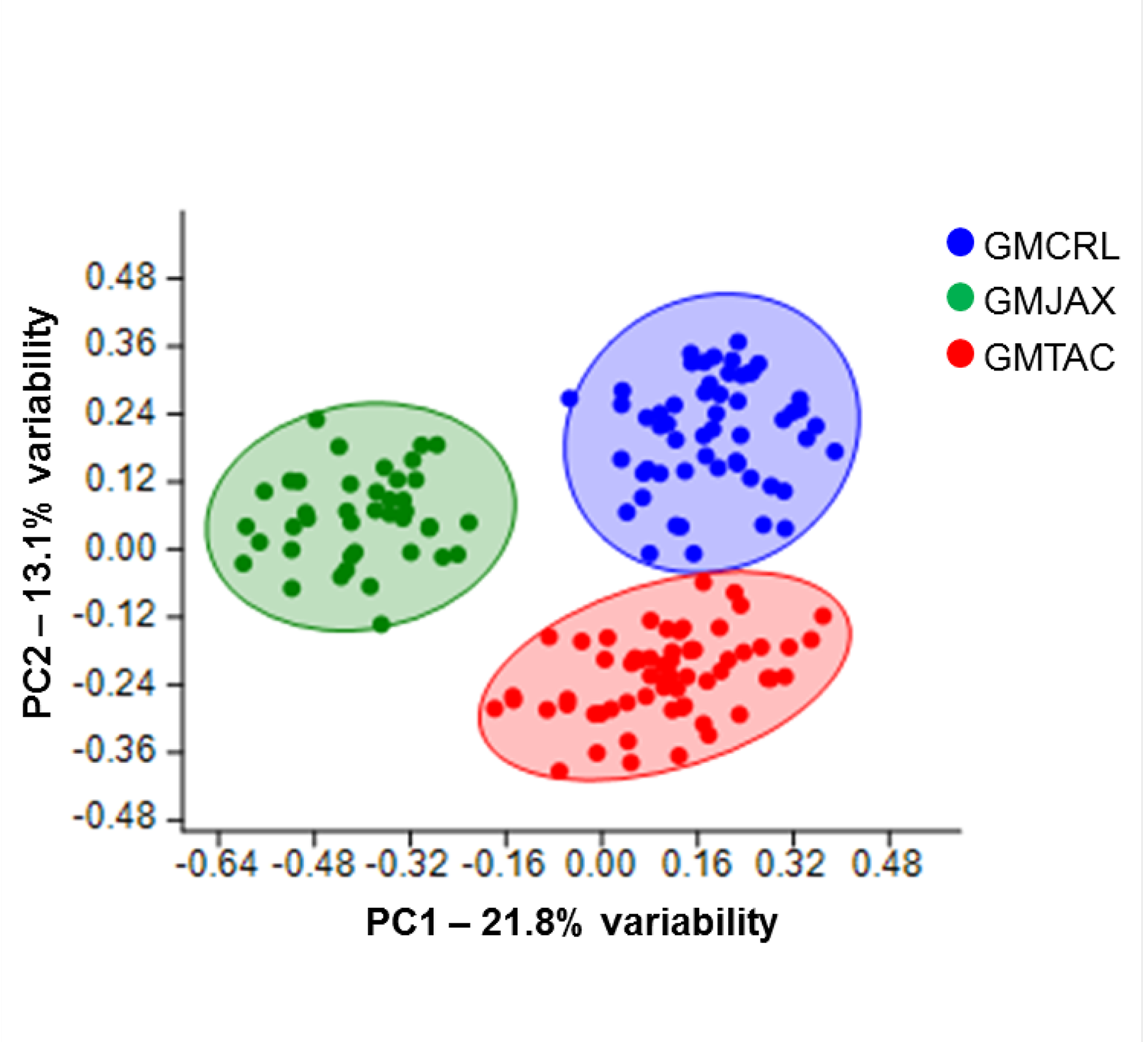

Supplement: Figure S3 — Principal component analysis of the gut microbiota (GM) of rederived CD1 mice. Representative samples from 1st and 2nd generation females at 8–10 weeks of age. n = 45–50. Blue circles = GMCRL; green circles = GMJAX; and red circles = GMTAC. Statistical significance determined using one-way PERMANOVA (p ≤ 0.05 significant). [file Image3.TIF]

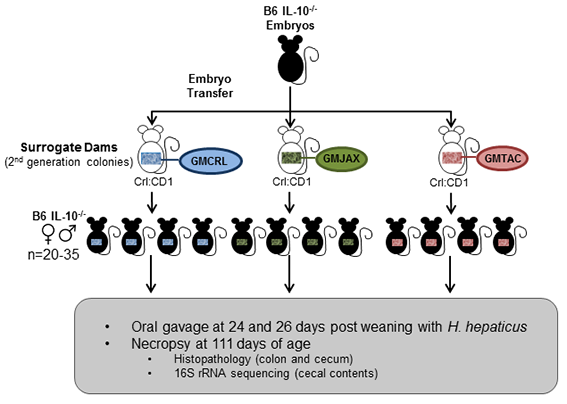

Supplement: Figure S4 — Experimental design used to generate IL-10−/− pups with different GI microbiota (GM). Schematic diagram showing embryo transfer scheme used to generate B6.129P2-Il10tm1Cgn/J (B6 IL-10−/−) pups rederived to second generation CD1GMCRL, CD1GMJAX, CD1GMTAC surrogate dams from previously established colonies. Pups were inoculated with H. hepaticus at 24 and 26 days of age and necropsied at 111 days of age. Cecal contents were submitted for sequencing and cecal and colonic disease evaluated. [file Image4.TIF]

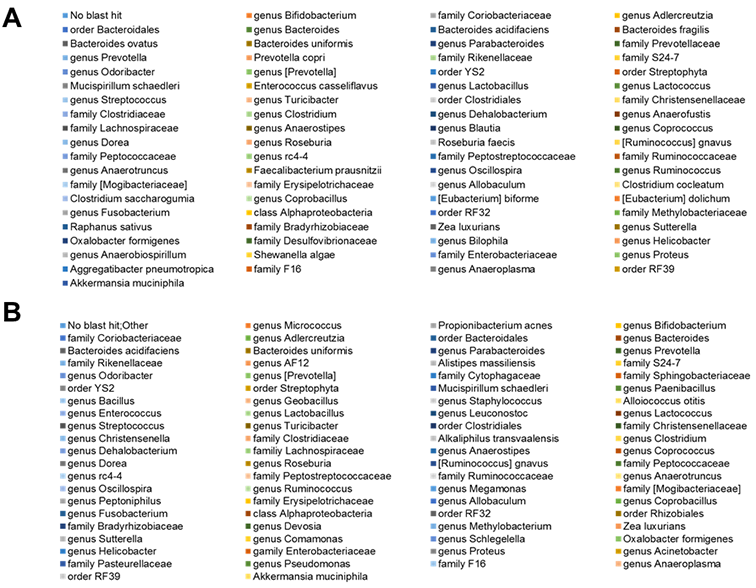

Supplement: Figure S5 — OTU relative abundance bar chart color legend. (A) OTU legend for Figures 4A,B. (B) OTU legend for Figure 8B. [file Image5.TIF]
