# Supplementary material for: Differing Complex Microbiota Alter Disease Severity of the IL-10−/− Mouse Model of Inflammatory Bowel Disease
Source: Front Microbiol. 2017 May 11;8:792. doi: 10.3389/fmicb.2017.00792 (PMC5425584; doi:10.3389/fmicb.2017.00792)
Supplement: Table S1 — Statistical comparison of the relative abundance of phyla between rederivation groups within the B6 IL-10−/− and C3H IL-10−/− models. P-value, mean fold change, and mean relative abundance are shown. Statistical significance and interactions of GM profile, sex, and OTU were determined using two-way ANOVA with Student Newman-Keuls post-test. Bold face values indicate statistical significance (p ≤ 0.01). [file Table1.DOCX]

**Table S1. Statistical comparison of the relative abundance of phyla between rederivation groups within the B6 IL-10^-/-^ and C3H IL-10^-/-^ models.**

| **Significant Phylum** | | **GMJAX relative to GMCRL** | | **GMJAX relative to GMTAC** | | **GMTAC relative to GMCRL** | | **GMCRL** | **GMJAX** | **GMTAC** |
| --- | --- | --- | --- | --- | --- | --- | --- | --- | --- | --- |
| **Mouse Model** | **Phylum** | ***p-*value** | **Mean Fold Change** | ***p-*value** | **Mean Fold Change** | ***p-*value** | **Mean Fold Change** | **Mean Relative Abundance (±SEM)** | **Mean Relative Abundance**  **(± SEM)** | **Mean Relative Abundance**  **(± SEM)** |
| B6 IL-10^-/-^ | *Deferribacteres* | **<0.001** | 0.256 | 0.248 | 0.509 | 0.013 | 0.510 | 0.021  ±0.003 | 0.005  ±0.001 | 0.01  ±0.002 |
|  | *Proteobacteria* | 0.580 | 1.24 | **0.01** | 0.540 | **0.01** | 0.764 | 0.014  ±0.002 | 0.017  ±0.003 | 0.032  ±0.007 |
|  | TM7 | **<0.001** | 0.176 | **0.003** | 0.447 | **0.008** | 0.379 | 0.015  ±0.003 | 0.003  ±0.0001 | 0.006  ±0.0001 |
| C3H IL-10^-/-^ | *Deferribacteres* | 0.386 | 1.923 | **<0.001** | 0.226 | **<0.001** | 9.904 | 0.008  ± 0.002 | 0.017  ±0.004 | 0.078  ±0.01 |
|  | *Proteobacteria* | **0.008** | 0.477 | 0.446 | 1.148 | **0.01** | 0.437 | 0.06  ±0.009 | 0.029  ±0.005 | 0.025  ±0.007 |
| B6 IL-10^-/-^ (CMTR) | *Deferribacteres* | 0.050 | **0.001** | **0.004** | 0.001 | 0.104 | 1.592 | 0.005  ±.0007 | 3.36E-06  ±1.85E-06 | 0.008  ±0.002 |
|  | *Proteobacteria* | **<0.001** | 3.195 | 0.033 | 1.624 | **0.006** | 1.967 | 0.005  ±0.0006 | 0.018  ±0.004 | 0.011  ±0.001 |
|  | TM7 | 0.120 | 0.294 | **0.006** | 0.163 | 0.047 | 1.802 | 0.009  ±0.001 | 0.003  ±0.0008 | 0.016  ±0.003 |
